# The impact of early neuraminidase inhibitor therapy on clinical outcomes in patients hospitalised with influenza A-related pneumonia: a multicenter, retrospective study

**DOI:** 10.1186/s12879-020-05322-x

**Published:** 2020-08-26

**Authors:** Liang Chen, Xiudi Han, Yan Li Li, Chunxiao Zhang, Xiqian Xing

**Affiliations:** 1 Department of Infectious Disease, Beijing Jishuitan Hospital, 4th Medical College of Peking University, Beijing, China; 2 Department of Pulmonary and Critical Care Medicine, Qingdao Municipal Hospital, Qingdao City, Shandong Province China; 3 Department of Infectious Diseases and Clinical Microbiology, Beijing Chao-Yang Hospital, Capital Medical University, Beijing, China; 4 Department of Pulmonary and Critical Care Medicine, Beijing Huimin Hospital, Beijing, China; 5 Department of Pulmonary and Critical Care Medicine, the 2nd People’s Hospital of Yunnan Province, Kunming City, Yunnan Province China

**Keywords:** Neuraminidase inhibitor, Influenza a, Community-acquired pneumonia, Clinical outcome

## Abstract

**Background:**

Guidelines emphasize prompt antiviral treatment in severe influenza patients. Although nearly a 50% of severe influenza present with pneumonia, the effect of early (≤ 2 days after illness onset) neuraminidase inhibitor (NAI) use on the clinical outcomes of influenza A-related pneumonia (FluA-p) has rarely been assessed. Furthermore, data about the administration of NAIs in the real-world management of Flu-p in China are limited.

**Methods:**

Data of patients hospitalised with FluA-p from five teaching hospitals in China from 1 January 2013 to 31 December 2018 were reviewed retrospectively. The impact of early NAI therapy on the outcomes in FluA-p patients, and the indications of early NAI administration by clinicians were evaluated by logistic regression analysis.

**Results:**

In total, 693 FluA-p patients were included. Of these patients, 33.5% (232/693) were treated early. After adjusting for weighted propensity scores for treatment, systemic corticosteroid and antibiotic use, a multivariate logistic regression model showed that early NAI therapy was associated with decreased risk for invasive ventilation [*odds ratio* (*OR*) 0.511, *95% confidence interval (CI)* 0.312–0.835, *p* = 0.007) and 30-day mortality (*OR* 0.533, *95% CI* 0.210–0.807, *p* < 0.001) in FluA-p patients. A multivariate logistic regression model confirmed early NAI use (*OR* 0.415, *95% CI* 0.195–0.858, *p* = 0.001) was a predictor for 30-day mortality in FluA-p patients and a positive rapid influenza diagnostic test was the only indication (*OR* 3.586, *95% CI* 1.259–10.219, *p* < 0.001) related to the prescription of early NAI by clinicians.

**Conclusions:**

Early NAI therapy is associated with better outcomes in FluA-p patients. Improved education and training of clinicians on the guidelines of influenza are needed.

## Background

Influenza is a highly contagious viral respiratory disease with global prevalence [1–4]. It is estimated that between 5 and 10% of the global population experience symptomatic influenza during an annual seasonal epidemic, including 3–5 million cases of severe illness and 290–650 thousand deaths from influenza-related respiratory illness [5, 6].

The neuraminidase inhibitors (NAIs), represented by oseltamivir, became the first anti-flu medication approved by the United Food and Drug Administration at the end of last century [7]. A randomized controlled trial (RCT) on uncomplicated outpatients within 48 h of symptom onset showed oseltamivir treatment decreased the duration of influenza by a median of 70 h and decreased patient-perceived severity of illness [8]. Subsequent observational studies suggested that severe influenza patients could also benefit from early (≤ 2 days after illness onset) NAI administration [9–11]. Therefore, the American and Chinese guidelines recommended early initiation of NAI therapy in the patients at high-risk of severe influenza [12, 13].

Influenza-related pneumonia (Flu-p), including primary viral and secondary bacterial pneumonia, which is mostly caused by influenza A, is the major cause of hospitalizations and deaths due to influenza [14–16]. Previous studies on NAIs and severe influenza have rarely targeted Flu-p, especially in the Chinese population. Furthermore, although the Chinese guidelines emphasize prompt antiviral treatment in severe influenza patients after the 2009 influenza pandemic, there is limited data about the administration of NAIs in the real-world management of Flu-p in China.

We carried out a multicenter, retrospective study with two aims: 1) to evaluate the impact of early NAI therapy on the clinical outcomes in adolescent and adult patients hospitalised with FluA-p; and 2) to investigate the indications of early NAI administration by Chinese clinicians.

## Methods

### Study design and population

We conducted a retrospective review of the data of all hospitalised patients who tested positive for influenza A virus RNA detected in respiratory specimens at five teaching hospitals in Beijing, Shandong and Yunnan Provinces during the period from 1 January 2013 to 31 December 2018 (details of the five centers are shown in Additional file 1: Appendix 1). From these cases, we retrieved the data of patients with laboratory-confirmed influenza A-related pneumonia (FluA-p) onset in the community.

The following exclusion criteria were applied: (1) age < 14 years; (2) pneumonia onset ≥48 h after admission and hospitalised within the last 28 days [17]; and (3) immunocompromized status [17].

### Data collection

The following data were retrospectively collected: demographic information, underlying disease (Additional file 1: Appendix 2), clinical manifestations, laboratory and radiological findings on admission, microbiological information, treatment (use of antiviral agents, antibiotics, corticosteroids and mechanical ventilation), clinical outcomes (admittance to the intensive care unit (ICU), 14-day and 30-day mortality).

### Study definitions

Patients with FluA-p were defined as patients with respiratory symptoms and a new pulmonary infiltrate on chest radiographs, combined with positive influenza virus A reverse transcription polymerase chain reaction (RT-PCR) tests during the influenza season in China. Early NAI use was defined as any NAI administered within 2 days after illness onset. Community-acquired respiratory co-infections resulting from coinfecting pathogens were identified using standard microbiologic procedures within the first 48 h after admission. The criteria for the definition of a community-acquired respiratory pathogen as the causative coinfecting etiology are shown in Additional file 1: Appendix file 3.

### Propensity scores for treatment

Propensity scores (Ps) for the likelihood of NAI treatment were calculated for each patient using a multivariate logistic regression model according to the report by Hirano and Imbens [18]. The following covariates were included in the study: age, sex, comorbidities (body mass index (BMI) ≥ 30 kg/m^2^, smoking, pregnancy, asthma, chronic obstructive pulmonary disease, cardiovascular disease, cerebrovascular disease, malignant solid tumor, chronic kidney disease and diabetes), and CURB-65 scores (confusion, urea, respiratory rate, blood pressure, age ≥ 65 years) as a measure of severity [19].

Weighted Ps (WPs) were calculated according to the following formula: WPs = Pt/Ps in patients with early NAI use, while WPs = (1-Pt)/(1-Ps) in the control patients, where Pt represents the proportion of patients with early NAI use in the total patients included in the study.

### Statistical analysis

All data were analyzed with SPSS 22.0 and measurement data were tested for normal distribution using the Kolmogorov–Smirnov test. Measurement data with normal distribution were reported as the mean ± standard deviation. Measurement data with non-normal distribution was reported as the median. The categorical variables were analyzed by the Chi-square test or Fisher’s exact test, and continuous variables were analyzed by Student’s *t*-test or the Mann–Whitney U-test. A *p*-value of < 0.05 was considered to indicate statistical significance and all probabilities were two-tailed. After adjusting for WPs for treatment, systemic corticosteroids and antibiotic use, a multivariate logistic regression model was used to evaluate the impact of early NAI therapy on the outcomes (invasive ventilation, 14-day mortality and 30-day mortality) in the FluA-p patients. Variables with a *p*-values of < 0.1 on univariate analysis were subsequently entered into the backward stepwise logistic regression analysis to identify risk factors for the 30-day mortality or the administration of NAI by the clinicians in FluA-p patients. Missing data in the covariates were included as a separate dummy category to allow for comparisons across the crude and adjusted analyses.

## Results

### Screening process

**Fig. 1:**
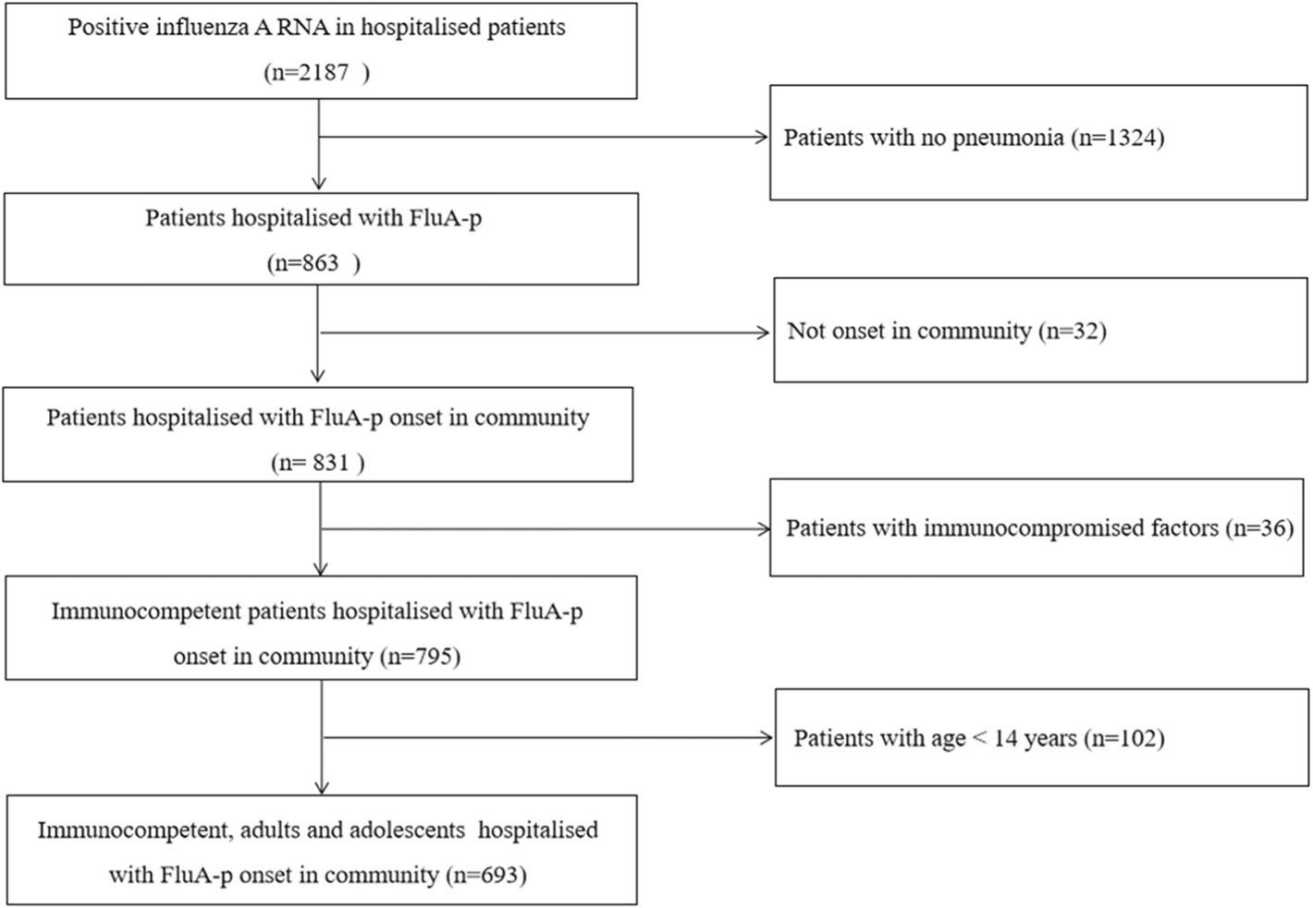
Patient screening algorithm for FluA-p

We screened 2187 hospitalised patients who tested positive for influenza A RNA. Overall, 693 immunocompetent adults and adolescent patients hospitalised with FluA-p were included in the final analysis (Fig. 1). Among these patients, 38.1% (264/693) were infected with A(H1N1) pdm09 and 11.0% (76/693) were infected with A(H3N2), while 50.9% (353/693) of patients were infected with an unclassified subtype. All patients received NAIs during the course of the disease, and 33.5% (232/693) were early NAI users.

### Overview of patients with FluA-p

Overall, the median age of the patients was 61.0 years and 65.1% (451/693) were males. Fifty-eight percent of patients (402/693) had at least one underlying disease. Only 4.6% (32/693) of patients had a consciousness disorder on admission. Respiratory rates ≥30 times/min and hypotension were identified in 17.5% (121/693) and 1.2% (8/693) of patients, respectively and 26.9% (172/639) of patients had pO_2_/FiO_2_ ≤ 250 mmHg. Multilobar infiltrate and pleural effusion in chest radiology was observed in 78.8% (546/693) and 16.6% (115/693) of patients, respectively (Table 1).

**Table 1:** The baseline characteristics of patients in the deceased and survival groups

Variables	Total (*n* = 693)	Deceased group (*n* = 136)	Survival group (*n* = 557)	*p*-value
Male (*n*,)	461 (66.5)	92 (67.6)	369 (66.2)	0.757
Age (median, IQR, years)	61.0 (36.0–73.0)	61.5 (28.0–76.0)	59.0 (36.0–72.0)	0.963
BMI ≥ 30 kg/m^2^ (*n*, %)^a^	48 (6.9)	0 (0.0)	48 (8.6)	**< 0.001**
Comorbidities (*n*, %)				
Hypertension	252 (36.4)	48 (35.3)	204 (36.6)	0.772
Cardiovascular disease^a^	136 (19.6)	48 (35.3)	88 (15.8)	**< 0.001**
Diabetes mellitus	92 (13.3)	14 (10.3)	78 (14.0)	0.253
Cerebrovascular disease	72 (10.4)	10 (7.4)	62 (11.1)	0.195
COPD^a^	40 (5.8)	3 (2.2)	37 (6.6)	**0.047**
Asthma	19 (2.7)	2 (1.5)	17 (3.1)	0.222
Chronic kidney disease	16 (2.3)	6 (4.4)	10 (1.8)	0.133
Malignant solid tumor	16 (2.3)	0 (0.0)	16 (2.9)	0.193
Smoking history^a^	243 (35.1)	68 (50.0)	175 (31.4)	**< 0.001**
Clinical and radiologic characteristics (*n*, %)				
Respiratory rates ≥30 times/min	121 (17.5)	25 (18.4)	96 (17.2)	0.752
Confusion^a^	32 (4.6)	32 (23.5)	0 (0.0)	**< 0.001**
SBP < 90 mmHg	8 (1.2)	0 (0.0)	8 (1.4)	0.338
Leukocytes > 10 × 10^9^/L ^a^	118 (17.0)	42 (30.9)	76 (13.6)	**< 0.001**
Lymphocytes < 0.8 × 10^9^/L ^a^	299/677 (44.2)	120 (88.2)	179/541 (33.1)	**< 0.001**
Hb < 100 g/L^a^	69 (10.0)	34 (25.0)	35 (6.3)	**< 0.001**
ALB < 35 g/L	58/639 (9.1)	12/131 (9.2)	46/508 (9.1)	0.970
BUN > 7 mmol/L^a^	183/685 (26.7)	97 (71.3)	86/549 (15.7)	**< 0.001**
BG > 11 mmol/L	48 (6.9)	8 (5.9)	40 (7.2)	0.397
Arterial pH < 7.35^a^	120/639 (18.8)	60/136 (44.1)	60/503 (11.9)	**< 0.001**
pO_2_/F_i_O_2_ ≤ 250 mmHg ^a^	172/639 (26.9)	28/136 (20.6)	144/503 (28.6)	0.061
Pleural effusion^a^	120 (17.3)	36 (26.5)	84 (15.1)	**< 0.001**
Early NAI use^a^ (*n*, %)^a^	232 (33.5)	60 (43.4)	172 (30.9)	**0.003**
Duration from illness onset to NAI use (days, median, IQR)^a^	3.0 (1.0–4.0)	2.5 (1.0–3.0)	3.0 (1.0–4.0)	**0.004**
Systemic corticosteroid use (*n*, %)^a^	132 (19.0)	60 (44.1)	72 (12.9)	**< 0.001**
Coinfection with other pathogens (*n*, %)^a^	265 (38.2)	84 (61.8)	181 (32.5)	**< 0.001**

^a^Variables cited in the table above were the candidates which were entered into the multivariate logistic regression model. The bolded values are *p*-values < 0.05, which represent significant differences between survival group and deceased group. *IQR* Interquartile range, *BMI* Body mass index, *COPD* Chronic obstructive pulmonary disease, *SBP* Systolic blood pressure, *Hb* Hemoglobin, *BG* Blood glucose, *ALB* Albumin, *BUN* Blood urea nitrogen, *pO*_*2*_*/FiO*_*2*_ Arterial pressure of oxygen/fraction of inspiration oxygen, *NAIs* Neuraminidase inhibitors; a: Neuraminidase inhibitor refers to any dose of oseltamivir, zanamivir or peramivir

As shown in Additional file 1: Appendix file 4, 38.2% (265/693) of FluA-p patients were coinfected with other community-acquired respiratory pathogens, with *Streptococcus pneumoniae* (33.2%) as the most common etiology, followed by *Klebsiella pneumoniae* (30.6%) and *Staphylococcus aureus* (20.4%)*.*

All patients were treated with antibiotics and 19% of patients (132/693) received systemic corticosteroids during hospitalisation. The 30-day mortality was 19.6% (136/693) (Table 1).

### The risk factors for 30-day mortality in FluA-p patients

According to the survival status at 30 days after admission, the patients were divided into survival and deceased groups. The baseline characteristics of the patients in the survival and deceased groups are shown in the Table 1. There were no significant differences in terms of age and sex between the two groups. Cardiovascular disease, smoking history, confusion, leukocytes > 10 × 10^9^/L, lymphocytes < 0.8 × 10^9^/L, Hb < 100 g/L, BUN > 7 mmol/L, arterial pH < 7.35 on admission, early use of NAIs and systemic corticosteroids during hospitalization were more common in the deceased group compared with the survival group, while BMI ≥ 30 kg/m^2^ and COPD were less common. Although more patients in the deceased group were coinfected with other pathogens, there was no significant differences in the spectrum of etiologies (Additional file 1: Appendix file 4).

The multivariate logistic regression model confirmed early NAI therapy [*odds ratio* (*OR*) 0.415, *95% confidence interval (CI)* 0.195–0.858, *p* = 0.001], cardiovascular disease (*OR* 3.189, *95% CI* 1.300–7.892, *p* < 0.001), smoking history (*OR* 3.294, *95% CI* 1.151–9.429, *p* < 0.001), lymphocytes < 0.8 × 10^9^/L (*OR* 4.080, *95% CI* 1.321–12.596, *p* < 0.001), BUN > 7 mmol/L (*OR* 2.158, *95% CI* 1.084–4.690, *p* < 0.001) and pO_2_/FiO_2_ ≤ 250 mmHg (*OR* 4.344, *95% CI* 2.050–9.203, *p* < 0.001) were independent risk factors for 30-day mortality in FluA-p patients (Table 2).

**Table 2:** The risk factors for the 30-day mortality in FluA-CAP patients

Variable	*OR* (*95% CI*)	*p-*value
Cardiovascular disease	3.189 (1.300–7.892)	< 0.001
Smoking history	3.294 (1.151–9.429)	< 0.001
Lymphocytes < 0.8 × 10^9^/L	4.080 (1.321–12.596)	< 0.001
BUN > 7 mmol/L	2.158 (1.084–4.690)	< 0.001
pO_2_/F_i_O_2_ ≤ 250 mmHg	4.344 (2.050–9.203)	< 0.001
Early NAI therapy	0.415 (0.195–0.858)	0.001

*OR* Odd ratio, *CI* Confidence interval

### The impact of early NAI use on the clinical outcomes of FluA-p patients

In the univariate analysis, early NAI therapy was associated with increased risk for 30-day mortality, but not with invasive ventilation or 14-day mortality.

After adjusting for WPs for treatment, systemic corticosteroid and antibiotic use, a multivariate logistic regression model showed that early use of NAI was associated with decreased risk of invasive ventilation (*OR* 0.511, *95% CI* 0.312–0.835, *p* = 0.007), 14-day mortality (*OR* 0.477, *95% CI* 0.124–0.744, *p* < 0.001) and 30-day mortality (*OR* 0.533, *95% CI* 0.210–0.807, *p* < 0.001) in FluA-p patients (Table 3).

**Table 3:** The impact of early NAI therapy on the clinical outcomes of FluA-CAP patients

Variable	Patients (*n*, %)		Univariate logistic analysis		Multivariate logistic analysis	
	Early NAI use group (*n* = 232)	Control group (*n* = 461)	*OR (95% CI)*	*p-*value	^a^a*OR (95% CI)*	*p-*value
Invasive ventilation	52 (22.4)	106 (23.0)	0.897 (0.612–1.317)	0.577	0.511 (0.312–0.835)	0.007
14-day mortality	32 (13.8)	48 (10.4)	1.377 (0.853–2.221)	0.190	0.477 (0.124–0.744)	< 0.001
30-day mortality	60 (25.9)	76 (16.5)	1.767 (1.205–2.592)	0.004	0.533 (0.210–0.807)	< 0.001

^a^Adjusted for weighted propensity scores for treatment, systemic corticosteroid and antibiotic use

**Fig. 2:**
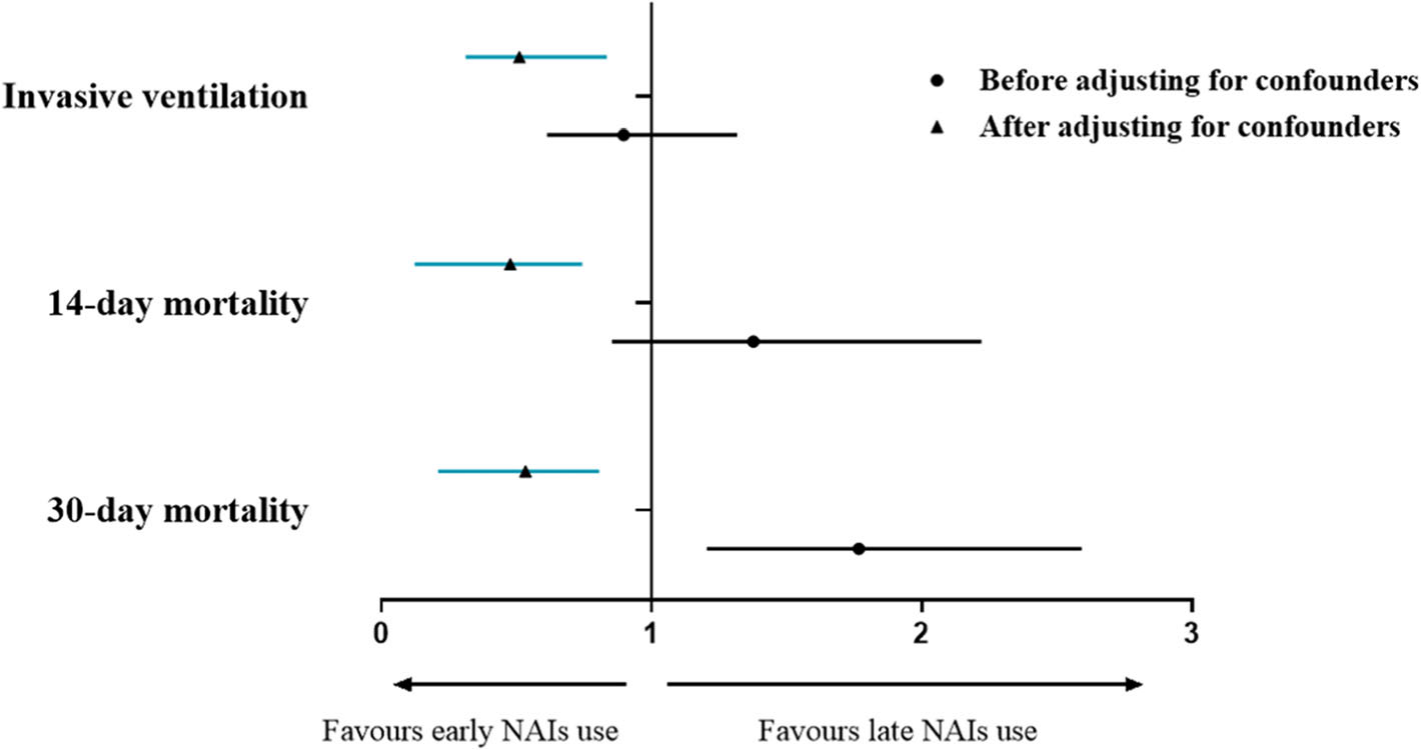
Forrest plot of the impact of early NAI therapy on the outcomes in the FluA-p patients. ▲: Adjusted for weighted propensity scores for treatment, systemic corticosteroid and antibiotic use

Figure 2 shows association between early NAI therapy and the outcomes of FluA-p patients before and after adjusting for confounders.

### The risk factors for early NAI administration prescribed by clinicians

Logistic regression analysis allowed us to further explore the factors predictive of NAI use in FluA-p patients. All the potential factors screened in the univariate analysis with *p* < 0.1 variables were included in the regression model: male, age ≥ 65 years old, cardiovascular disease, diabetes mellitus, cerebrovascular disease, COPD, asthma, chronic kidney disease, malignant solid tumor, axillary temperature ≥ 38 °C, cough, chest pain, confusion, SBP < 90 mmHg, leukocytes > 10 × 10^9^/L, leukocytes < 4.0 × 10^9^/L, lymphocytes < 0.8 × 10^9^/L, PO_2_/FiO_2_ ≤ 250 mmHg, pleural effusion and positive for RIDTs (Additional file 1: Appendix file 5).

A multivariate logistic regression model suggested that positive RIDTs (*OR* 6.504, *95% CI* 1.671–25.323, *p* = 0.007) was the only predictor for early NAI administration by clinicians in the FluA-p patients (Table 4).

**Table 4:** The risk factors for early NAI administration by the clinicians

Variable	*p*-value	*OR* (*95% CI*)
Positive for RIDTs	< 0.001	3.586 (1.259–10.219)

*RIDTs* Rapid influenza diagnostic tests

**Fig. 3:**
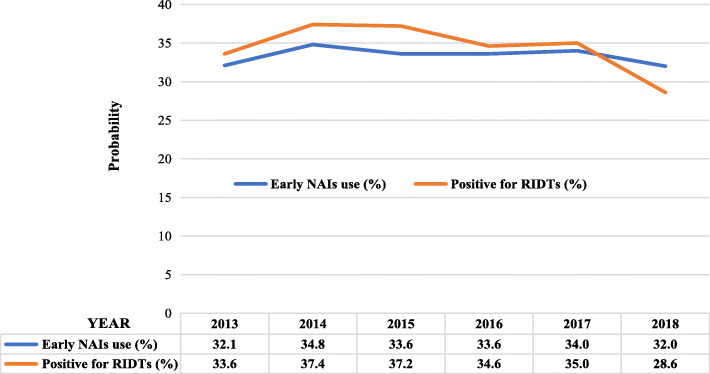
The proportion of early NAI treatment and RIDT-positive FluA-p patients treated during 2013–2018 in China

Figure 3 shows similar proportions of early NAI administration and positive RIDT in FluA-p patients treated during the study period, both of which fluctuated up and down by 30%.

## Discussion

Our study had two important findings. First, compared with late use of NAI, early initiation of NAI therapy was associated with better outcomes in FluA-p patients and second, in real-world management of FluA-p patients, positive RIDT was the only indication for early NAI administration by the Chinese clinicians.

Previous studies have shown that the mortality due to influenza-related pneumonia ranged from 5 to 50% [20, 21]. In our study, the 30-day mortality rate was 19.6%, which was in accordance with these previous reports. A RCT is considered to be the most effective approach to the evaluation of the effect of a medication [22]. The World Health Organization have formally endorsed the use of NAIs in high-risk or severely ill patients with influenza; therefore, RCTs in populations such as patients with influenza-related pneumonia are unlikely due to ethical concerns. Unlike RCTs, observational studies are subject to selective bias, and a large number of confounders may have a significant impact on the outcomes [23, 24]. Patients with more severe disease may tend to receive treatment earlier (or later), so the therapeutic effect will be exaggerated (or attenuated). For example, in our study, more deceased patients received early NAI treatment, and the period from disease onset to the initiation of NAI treatment was slightly shorter. After control for other confounders (e.g., obesity, cardiovascular disease, illness severity at admission and systemic corticosteroid use), the direction of the association between early use of NAI and mortality changed. To minimize the selective bias, we used two methodologies to control for the potential confounders. Both sets of results confirmed the association of early NAI therapy with better outcomes, with very similar *OR* values for mortality. Muthuri [25] conducted a meta-analysis of 20,634 severely ill patients with (H1N1) pdm09 infections from nine centers all over the world using individual data rather than group data for greater accuracy. This analysis revealed that early NAI therapy was associated with decreased risk of invasive ventilation [hazard ratio (*HR*) 0.68, *95% CI* 0.54–0.85) and mortality (*HR* 0.70, *95% CI* 0.55–0.88) in influenza-related pneumonia compared with late NAI therapy. In fact, although few studies have focused on influenza-related pneumonia, most showed early NAI use improved the clinical outcomes in severe cases of influenza, including reducing the incidence of complications and decreasing the mortality rate [26–28]. A study suggested a potential benefit of NAI treatment even 48 h after the onset of symptoms in the very sick patients [26].

Choi [29] conducted a retrospective study of 508 patients hospitalised with severe influenza during 2010 and 2011, with 28.3% of the patients complicated by pneumonia. The results of this study showed that early NAI therapy was associated with decreased risk of ICU admission, but not with the in-hospital mortality; however, the timing of NAI use might be a risk factor for ICU admittance, an association that was not analyzed. In addition, 28.1% of patients in this study were infected with influenza B, and the authors speculated that the discrepancy with the results of others was due to the fact that NAIs are less effective against influenza B compared to influenza A. However, two other studies showed that patients with severe influenza B also benefited from NAI therapy [10, 30].

Although RT-PCR detection of RNA has become the standard diagnostic test for influenza infection, its extensive use is limited by many factors, such as staff expertise, equipment maintenance, testing procedures and many other aspects of laboratory operations, in addition to the cost [31, 32]. Rapid influenza diagnostic tests (RIDTs) have three main advantages of low cost, technical simplicity and rapid results that can be obtained in 10 to 15 min, which is significantly faster than the period of 1 h or more required for RT-PCR methods [33, 34]. However, RIDTs are less sensitive than RT-PCR and require 10^4^ to 10^6^ infectious influenza particles for a positive result [35]. Previous reports showed more than 40% false-negative results obtained by RIDTs [36, 37]. During epidemics, the Chinese guidelines on influenza recommend that the primary diagnosis of influenza infection is dependent on influenza-like illness (ILI) and some studies have shown the accuracy of ILI is between 60 and 70% compared with RT-PCR methods in the diagnosis of influenza [38, 39]. The guidelines also recommend early identification and antiviral treatment in patients at high-risk of severe influenza, according to host factors (e.g., age, comorbidities, and pregnancy) and clinical presentations (e.g., consciousness disorder, respiratory distress, hypotension, and hypoxemia).

In our study, the patients receiving early NAI therapy were younger, with a lower frequency of underlying disease, and the indexes of illness severity, such as shortness of breath, confusion and oxygenation status, were not worse than those of the control patients. A multivariate logistic regression analysis showed that a positive RIDT was the only indication of early NAI administration by the clinicians. The proportions of patients with a positive RIDT and receiving early NAI therapy were very similar, accounting for nearly one-third of the total patients each year. Previous reports revealed that between 10 and 60% of severe influenza patients received early NAI treatment [40–42]. Data for patients’ delay in visiting the doctor and unavailability of medication, which could not be collected and evaluated in our retrospective study; however, our results indicated that over-reliance on the results of RIDTs in influenza diagnosis, and ignorance of the clinical characteristics and host factors that identify patients at high-risk of severe influenza contributed to two-thirds of influenza pneumonia patients missing the chance of receiving early NAI therapy. As all the participating centers were teaching hospitals, we thought this phenomenon might be universal and representative.

There are some limitations specific to our study that should be mentioned. First, in addition to the retrospective nature of this study, some missing data might limit the accuracy of our results. Second, more than one-third of the patients had not undergone influenza subtype testing and other respiratory tract viruses were not routinely detected; thus, we could not exclude coinfection with other viruses. Third, a few studies indicated that the antiviral susceptibility profile of influenza A viruses in immunocompromized patients was not the same as that in immunocompetent hosts [43, 44]. Therefore, the conclusions of our research should be assessed prudently before being applied in immunocompromized patients. Finally, due to the retrospective study design, vaccination data could not be retrieved to allow adjustment for this potential in the logistic regression analysis.

## Conclusions

Our study confirmed that early initiation of NAI therapy is associated with better outcomes in FluA-p patients, which supported the recommendations for NAI use in the current guidelines. Our findings also suggest that better education and training of clinicians on current influenza guidelines in China are needed to improve the management of severe influenza.

## Supplementary information

**Additional file 1 Appendix 1:** Details of participating centers. **Appendix 2.** Definition of underlying diseases. **Appendix 3.** Definition of microbiological criteria of coinfected with other pathogens. **Appendix 4**. coinfection with other community-acquired pathogens. **Appendix 5.** The comparison of patients in the early NAIs use group and the control group.

## Data Availability

The datasets used and/or analyzed during the current study is available from the corresponding author on reasonable request.

## Abbreviations

Flu-p: Influenza-related pneumonia
NAI: Neuraminidase inhibitor
RCT: Randomized controlled trial
OR: Odds ratio
HR: Hazard ratio
95% IC: 95% Interval confidence
FluA-p: Influenza A-related pneumonia
RT-PCR: Reverse transcription-polymerase chain reaction
IQR: Interquartile range
BMI: Body mass index
COPD: Chronic obstructive pulmonary disease
SBP: Systolic blood pressure
Hb: Hemoglobin
BG: Blood glucose
ALB: Albumin
BUN: Blood urea nitrogen
PH: Hydrogen ion index
pO_2_/FiO_2_: Arterial pressure of oxygen/fraction of inspiration oxygen
ICU: Intensive care unit
RIDTs: Rapid influenza diagnostic tests
ILI: Influenza-like illness
