# Supplementary material for: The impact of early neuraminidase inhibitor therapy on clinical outcomes in patients hospitalised with influenza A-related pneumonia: a multicenter, retrospective study
Source: BMC Infect Dis. 2020 Aug 26;20:628. doi: 10.1186/s12879-020-05322-x (PMC7447583; doi:10.1186/s12879-020-05322-x)
Supplement: Supplementary file 1 — Additional file 1 Appendix 1: Details of participating centers. Appendix 2. Definition of underlying diseases. Appendix 3. Definition of microbiological criteria of coinfected with other pathogens. Appendix 4. coinfection with other community-acquired pathogens. Appendix 5. The comparison of patients in the early NAIs use group and the control group. [file 12879_2020_5322_MOESM1_ESM.docx]

**Appendix 1: Details of participating centers**

| **Name of the hospital** | **Province, city** | **Teaching Hospital** | **Beds** | **Staffs of Clinical Microbioloy Lab** |
| --- | --- | --- | --- | --- |
| Beijing Jishuitan Hospital, | Beijing | Yes | 1500 | 10 |
| Beijing Chao-Yang Hospital | Beijing | Yes | 1400 | 11 |
| **the 2nd People’s Hospital of Yunnan Province** | **Kunming,**  **Yan’an** | Yes | 1302 | 4 |
| **Qingdao Municipal Hospital** | ShanDong,  Qingdao | Yes | 1200 | 4 |
| Beijing Huimin Hospital | Beijing | Yes | 1000 | 2 |

**Appendix 2 Definition of underlying diseases**

1. Smoking was defined as cigarette smokers of 10 cigarettes/d during at least the previous year；
2. Hypertension was defined as systolic blood pressure≥140mmHg and /or diastolic blood pressure ≥ 90mmHg in resting status;
3. Chronic pulmonary disease was defined as: persistent airflow limitation, FEV_1_ / FVC < 70% post bronchodilator;
4. Asthma was defined by the history of respiratory symptoms such as wheeze, cough that varied over time and intensity, together with variable respiratory airway limitation;
5. Cardiovascular disease included coronary heart disease and chronic congestive heart failure;
6. Coronary heart disease included angina pectoris, myocardial infarction, ischemic cardiomyopathy;
7. Chronic congestive heart failure was defined as cardiomegaly and ejection fraction ≤ 40%;
8. Cerebrovascular diseases included transient ischemic attack, cerebral hemorrhage, subarachnoid hemorrhage, cerebral infarction;
9. Diabetes mellitus: included diabetes mellitus type 1 and diabetes mellitus type 2, not included impaired glucose tolerance and impaired fasting glycaemia;
10. Chronic kidney disease included diabetic nephropathy, hypertensive renal damage, chronic glomerulonephritis, chronic pyelonephritis, lupus nephritis, IgA nephropathy, nephrotic syndrome, hereditary kidney disease, etc;
11. Immunosuppressive therapy: was defined as systmetic glucocorticosteroid (such as prednisone ≥ 10mg/d for more than 3 weeks in the last month); cyclosporine or azathioprine use within 3 months, and methotrexate use ≥ 12.5mg/week within 3 months; biological modifiers such as etanercept and infiximab within 3 weeks.
12. Immunocompromised status included HIV(+)，chemotherapy/radiotherapy within 6 months, immunosuppressive therapy, organ/bone marrow transplantation, splenectomy, hematological neoplasms.

**Appendix 3 Definition of microbiological criteria of coinfected with other pathogens**

1. Positive urinary antigen for *Legionella pneumophila*;
2. Positive urinary antigen for *Streptococcus pneumoniae*;
3. Positive bacterial culture from blood or plural fluid except for coagulase negative *Staphylococcus spp*.;
4. Paired sera with a fourfold or more increase in the titers of antibodies to *Mycoplasma pneumoniae* (MP), *Chlamydia pneumonia*, *L pneumophila or* respiratory viruses ( Parainfluenza, Adenovirus, Respiratory syncytial virus)*.* Or Serum IgM antibody (MIF) ≥ 1:16 for *Chlamydia pneumonia*;
5. Detection of respiratory virus in sputum/bronchoalveolar lavage (BALF)/throat swabs by Realtime-PCR according to manufacturer’s instructions, including respiratory syncytial virus (RSV) types A and B, parainfluenza virus (PIV) types 1, 2, 3 and 4, rhinovirus (HRV), enterovirus (EV), coronavirus (hCoV) types 229E, NL63, OC43 and HKU1, parapneumovirus (hMPV), and adenovirus (AdV), bocavirus;
6. Bacteria isolated form purulent sputum (defined as an adequate quality sputum sample with > 25 leukocytes and < 10 epithelial cells per × 100 magnification field) with compatible findings of Gram staining;
7. Detection of *Mycoplasma pneumoniae* (MP), *Chlamydia pneumonia* or *L pneumophila* in sputum/BALF/throat swabs by Realtime-PCR;
8. serum IgM antibody positive for *Mycoplasma pneumoniae* (MP), or Serum IgG antibody (MIF) ≥ 1:512 for *Chlamydia pneumonia;*
9. Invasive pulmonary aspergillosis were diagnosed in accordance with the revised definitions of invasive fungal diseases from the European Organization for Research and Treatment of Cancer and the Mycoses Study Group Education and Research Consortium [1].

References

1.Donnelly JP, Chen SC, Kauffman CA, et al. Revision and Update of the Consensus Definitions of Invasive Fungal Disease From the European Organization for Research and Treatment of Cancer and the Mycoses Study Group Education and Research Consortium. Clin Infect Dis. 2019, pii: ciz1008.

**Appendix 4 coinfection with other community-acquired pathogens**

| Variable | Total  (*n* = 693) | Deceased group  (*n* = 136) | Survival group (*n* = 557) | *p*-value |
| --- | --- | --- | --- | --- |
| Coinfection (*n*, %) | 265 (38.2) | 84 (61.8) | 181 (32.5) |  |
| Pathogens (*n*, %) |  |  |  |  |
| *Klebsiella pneumoniae* | 81 (30.6) | 25 (29.8) | 56 (30.9) | 0.847 |
| *Streptococcus pneumoniae* | 88 (33.2) | 25 (29.8) | 63 (34.8) | 0.417 |
| *Staphylococcus aureus* | 54 (20.4) | 21 (23.8) | 33 (18.8) | 0.203 |
| *Haemophilus influenzae* | 17 (6.4) | 6 (7.1) | 11 (6.1) | 0.742 |
| *Pseudomonas aeruginosa* | 12 (4.5) | 4 (4.8) | 8 (4.4) | 1.000 |
| *Klebsiella acidogens* | 7 (2.6) | 2 (2.4) | 5 (2.8) | 1.000 |
| *Acinetobacter* | 5 (1.9) | 2 (2.4) | 3 (1.7) | 1.000 |
| *Proteus spp.* | 3 (1.1) | 1 (1.2) | 2 (1.1) | 1.000 |
| *Stenotrophomonas maltophilia* | 3 (1.1) | 0 (0.0) | 3 (1.7) | 0.554 |
| *Escherichia coli* | 2 (0.8) | 0 (0.0) | 2 (1.1) | 1.000 |
| *Aspergillus spp.* | 2 (0.3) | 1 (1.2) | 1 (0.6) | 0.534 |
| *Citrobacter spp.* | 1 (0.3) | 1 (1.2) | 0 (0.0) | 0.317 |

4 deceased patients and 6 survival patients were coinfected with ≥ 2 pathogens

Appendix 5 The comparison of patients in the early NAIs use group and the control group

| Variables | Early NAIs use group (*n* = 232) | Control group  (*n* = 461) | *p* value |
| --- | --- | --- | --- |
| Male (*n*, %)^#^ | 184 (79.3) | 277 (60.1) | **< 0.001** |
| Age ≥ 65 years old^#^ | 48 (20.9) | 224 (48.6) | **< 0.001** |
| BMI ≥ 30 kg/m^2^ (*n*, %) | 16 (6.9) | 32 (6.9) | 0.982 |
| Pregnancy | 0 (0.0) | 8 (1.7) | 0.101 |
| Comorbidities (*n*, %) | 80 (34.5) | 283 (60.7) | **< 0.001** |
| Cardiovascular disease^#^ | 16 (6.9) | 120 (26.0) | **< 0.001** |
| Diabetes mellitus^#^ | 0 (0.0) | 92 (20.0) | **< 0.001** |
| Cerebrovascular disease^#^ | 40 (17.2) | 32 (5.0) | **< 0.001** |
| COPD^#^ | 23 (9.9) | 17 (3.7) | **0.001** |
| Asthma^#^ | 13 (5.6) | 6 (1.3) | **0.001** |
| Chronic kidney disease^#^ | 0 (0.0) | 16 (3.5) | **< 0.001** |
| Malignant solid tumor^#^ | 0 (0.0) | 16 (3.5) | **0.004** |
| Clinical and radiologic features (*n*, %) |  |  |  |
| Axillary Temperature ≥ 38℃^#^ | 232 (100.0) | 429 (93.1) | **< 0.001** |
| Running nose | 56 (24.1) | 99 (21.5) | 0.427 |
| Pharyngalgia | 56 (24.1) | 107 (23.2) | 0.786 |
| Cough^#^ | 232 (100.0) | 447 (97.0) | **0.017** |
| Chest pain^#^ | 0 (0.0) | 32 (6.9) | **< 0.001** |
| Confusion^#^ | 0 (0.0) | 32 (6.9) | **< 0.001** |
| Respiratory rates ≥ 30 times/min | 33 (14.2) | 88 (19.1) | 1.000 |
| SBP < 90 mmHg ^#^ | 8 (3.4) | 0 (0.0) | **< 0.001** |
| Leukocytes > 10×10^9^/L ^#^ | 16 (6.9) | 102 (22.1) | **< 0.001** |
| Leukocytes < 4.0×10^9^/L^#^ | 80 (34.5) | 64 (13.9) | **< 0.001** |
| Lymphocytes < 0.8×10^9^/L^#^ | 143/232 (61.6) | 156/445 (35.1) | **< 0.001** |
| PO_2_/FiO_2_ < 250 mmHg^#^ | 40/216 (18.5) | 132/423 (31.2) | **0.001** |
| Multilobar infiltrates | 176 (75.9) | 370 (80.3) | 0.181 |
| Pleural effusion^#^ | 72 (31.0) | 43 (9.3) | **< 0.001** |
| Positve for RIDTs (*n*, %)^#^ | 136 (58.6) | 100 (21.7) | **< 0.001** |

#Variables cited in the table above were the candidates which were entered into the multivariate logistic regression model. The bolded values are p-values < 0.05, which represent significant differences between early NAIs use group and control group. BMI: body mass index; COPD: chronic obstructive pulmonary disease; SBP: systolic blood pressure; PO_2_/FiO_2:_ arterial pressure of oxygen / fraction of inspiration oxygen_;_ RIDTs: rapid influenza diagnostic tests
